# Potential scenarios for the progression of a COVID-19 epidemic in the European Union and the European Economic Area, March 2020

**DOI:** 10.2807/1560-7917.ES.2020.25.9.2000202

**Published:** 2020-03-05

**Authors:** Helen C Johnson, Céline M Gossner, Edoardo Colzani, John Kinsman, Leonidas Alexakis, Julien Beauté, Andrea Würz, Svetla Tsolova, Nick Bundle, Karl Ekdahl

**Affiliations:** 1 European Centre for Disease Prevention and Control, Solna, Sweden; 2 These authors contributed equally

**Keywords:** SARS-CoV-2, coronavirus, pandemic, COVID-19, Europe, preparedness

## Abstract

Two months after the emergence of severe acute respiratory syndrome coronavirus 2 (SARS-CoV-2), the possibility of established and widespread community transmission in the European Union and European Economic Area (EU/EEA) is becoming more likely. We provide scenarios for use in preparedness for a possible widespread epidemic. The EU/EEA is moving towards the ‘limited sustained transmission’ phase. We propose actions to prepare for potential mitigation phases and coordinate efforts to protect the health of citizens.

On 31 December 2019, the Chinese health authorities reported a cluster of 27 pneumonia cases of unknown aetiology in Wuhan city, Hubei Province, China. The causative agent was later identified as a novel coronavirus named severe acute respiratory syndrome coronavirus 2 (SARS-CoV-2). In the first weeks of 2020, the number of cases increased and cases were soon reported outside of China. 

The aim of this paper was to provide a general scenario planning framework that can be used by European Union and European Economic Area (EU/EEA) countries in preparation for a possible widespread epidemic of coronavirus disease 2019 (COVID-19).

## Baseline situation for the scenarios

As at 1 March, 87,024 cases and 2,979 associated deaths have been reported worldwide [1]. The vast majority of the deaths (96%) have been reported in China [1]. Despite the high number of cases reported globally, estimates of the severity pyramid of disease and case fatality rate remain very uncertain; one large study conducted in China estimated that the majority (81%) of the cases were mild (i.e. non-pneumonia or mild pneumonia), 14% were severe (e.g. with dyspnoea) and 5% were in a critical condition (i.e. respiratory failure, septic shock and/or multiple organ dysfunction/failure) [2]. The case fatality ratio was 2.3% [2].

Despite extraordinary containment measures implemented in China, including the enforced lockdown of several cities and closures of schools, the virus has spread throughout the country and internationally [2]. It is too early to predict with any certainty the epidemiological developments over the coming weeks, but the possibility of widespread community transmission becoming established throughout the EU/EEA is becoming increasingly likely. 

We performed a strengths, weaknesses, opportunities and threats (SWOT) analysis (Box), which guided the development of scenarios for the potential progression of the epidemic in the EU/EEA. For this SWOT analysis, points that were directly linked to the EU/EEA were classified as strengths or weaknesses; points that were not directly linked to the EU/EEA but (potentially) affecting the EU/EEA were categorised as opportunities or threats.

Box **Strengths, weaknesses, opportunities and threats in relation to COVID-19, EU/EEA, 2020**

**Strengths**

- Low incidence rates of COVID-19 in the EU/EEA region outside the most affected areas allow time to learn about epidemiology and the effectiveness of control measures from the global experience.
- Response efforts are coordinated at the EU/EEA level, enabling harmonisation of surveillance and epidemiological assessment.
- Information about cases within the EU/EEA can be gathered and exchanged rapidly and securely using tools such as the Early Warning and Response System (EWRS) and The European Surveillance System (TESSy).
- All EU/EEA countries evaluate that they have the laboratory capacity to detect cases [6].
- Under scenario 1 and 2 (see below), EU/EEA countries are expected to have the hospital capacity and human resources required to isolate and care for cases and to conduct contact tracing.
- Well-functioning influenza surveillance systems in the EU/EEA can rapidly be adapted to detect SARS-CoV-2 infections of differing severity.
- All EU/EEA countries have pandemic preparedness plans that could be adapted and activated should the epidemiological situation deteriorate.

**Weaknesses**

- Large uncertainty remains regarding the severity of disease, case fatality ratios and risk groups.
- Mild and asymptomatic cases are likely to be undetected [7] but may contribute to transmission [8].
- Pressure on healthcare systems because of seasonal influenza may reduce the surge capacity needed to cope with the additional demand from COVID-19 cases in the EU/EEA.
- Entry screening is not effective because the majority of cases would be in the incubation period and therefore undetected.
- Supply chains for personal protective equipment and medicines may not be resilient to the disruption of a global outbreak.
- The capacity to isolate cases can be stretched only to a certain extent.
- The transmission potential of SARS-CoV-2 through substances of human origin remains unknown.
- Concentrated transmission during mass gathering events in the EU/EEA would hamper containment efforts.
- Co-occurrence with an outbreak of, for example, West Nile fever or chikungunya virus infection would add pressure on public health authorities. Similarly, ongoing prevention programmes (e.g. measles vaccination campaigns) may be affected.

**Opportunities**

- Considerable containment efforts in China in January and February appear to have limited transmission and therefore exportation.
- Global surveillance and communication efforts improve knowledge about the virus, the disease and transmission patterns.
- If SARS-CoV-2 demonstrates seasonality like influenza and other respiratory tract viruses, a decline in cases during summer would provide time to prepare for the following transmission season.

**Threats**

- It is uncertain how long the authorities in the most affected countries will be able to sustain the present containment measures.
- Large outbreaks now taking place outside of China increase the rate of importation into and within the EU/EEA, challenging the containment strategy currently in place.
- Some countries are reporting fewer importations than would be inferred from travel volumes, suggesting that circulation of the virus is more widespread than it appears from case detection and a pandemic may occur sooner than expected [9].
- Globalisation of trade and frequent international travel will facilitate the spread of the virus.
- Vaccines and antiviral treatments are under development but may not be widely available for several months or even years.
- The outbreak could cause substantial social, political and economic disruption also in parts of the world not directly affected by the virus.

COVID-19: coronavirus disease 2019; EU/EEA: European Union/European Economic Area; SARS-CoV-2: severe acute respiratory syndrome-related coronavirus 2.

## Potential scenarios for the progression of the COVID-19 epidemic

Based on epidemiological factors, we characterised three sequential scenarios for the spread of SARS-CoV-2 in the EU/EEA (Figure). The third scenario is divided in two sub-scenarios based on the impact on the healthcare system. The scenarios are: (1) short, sporadic chains of transmission, (2) localised sustained transmission, (3a) widespread sustained transmission with increasing pressure on the healthcare system and (3b) widespread sustained transmission with overburdened healthcare system. These scenarios are presented together with suggested control measures to limit the impact of the epidemic. It should be noted that at different points in time, different countries may find themselves in different scenarios. Some countries may skip one scenario to progress directly the following one.

**Figure:**
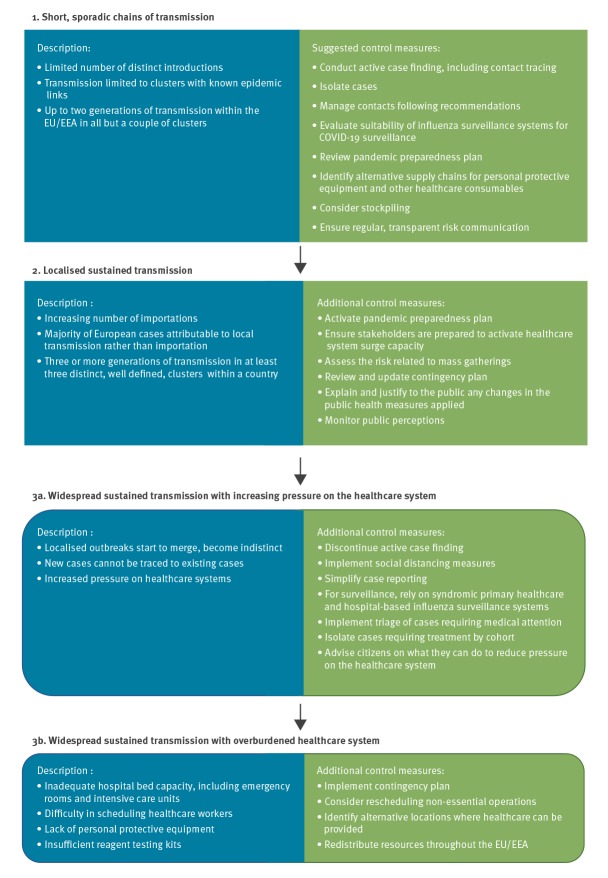
Scenarios for the potential spread and impact of COVID-19 in the EU/EEA, with suggested actions for containment and mitigation, March 2020

COVID-19: coronavirus disease 2019; EU/EEA: European Union/European Economic Area; SARS-CoV: severe acute respiratory syndrome coronavirus.

Up until 23 February, the number of cases in the EU/EEA was low and cases in Europe were either imported or part of well-defined transmission chains in Germany and France. Since the beginning of the outbreak, the response of countries to SARS-CoV-2 has been to limit virus importation and to contain clusters of cases as swiftly as possible. Those response measures, with the support of the measures taken in China, were initially effective in limiting the introduction of the virus to the EU/EEA. Besides preventing incident cases, they have delayed a larger outbreak, allowing time to review and implement preparedness measures, and also avoiding the peak influenza season. However, by 27 February, 92% (424/463) of cases reported in the EU/EEA had been locally acquired. The majority of those locally acquired cases (93%) have been found in Italy and from there, seeding events occurred in other EU/EEA countries. Considering the high number of cases reported within the EU/EEA, the European Centre for Disease Prevention and Control (ECDC) has since 28 February stopped distinguishing imported from locally acquired cases. While some EU/EEA countries are under the first scenario ‘short, sporadic chains of transmission’, others have reached or are about to reach the third scenario ‘Widespread sustained transmission with increasing pressure on the healthcare system’. The situation in the EU/EEA may change rapidly and countries may rapidly move from one scenario to the next at any time.

In *Scenario 1: Short, sporadic chains of transmission*, learning about the epidemiology of the virus is paramount. While the number of cases remains small, active case finding, including contact tracing, should be conducted. Swabbing of asymptomatic individuals may be considered. It is of paramount importance that contacts of cases are properly managed [3]. Cases should be isolated to avoid further transmission. The potential adaptation of influenza surveillance systems should be evaluated. It is now also advisable for countries to review their pandemic preparedness plan, including healthcare system surge capacity and plans for business and essential service continuity. Alternative supply chains should be identified for personal protective equipment and other healthcare consumables; stockpiling should be considered as supply chains may later be disrupted. Risks should be communicated in a transparent and consistent way to stakeholders and to the general public, according to the unfolding epidemiological situation. Messages should include the actions being taken with acknowledgement of uncertainty.

With the continuous introduction of SARS-CoV-2 and the ability of the virus to rapidly spread, the EU/EEA is about to enter *Scenario 2: Limited sustained transmission*. Countries should consider activating their pandemic preparedness plan. With a rising number of cases, resources may become stretched but detailed case histories, lists of contacts and samples for diagnostic testing should still be obtained, where possible. These data will give valuable insight into the epidemiology of the virus and will be essential in planning for further progression of the outbreak. While cases are concentrated in clusters, measures could be taken to boost capacity by transferring healthcare resources and staff from other locations. In preparation for the following scenarios, contingency plans should be reviewed and updated. Risk assessments before mass gatherings should consider their potential role in amplifying transmission of the virus. In this phase, there may be increasing concern among the population, particularly if a high level of uncertainty persists regarding disease severity. Risk communication messages should include clear justifications for any changes to the public health measures being implemented, as well as the critical importance of adherence to any such measures that may restrict personal freedom, such as quarantine or self-isolation. In addition, public perception should be monitored, regarding the outbreak itself but also the response, so that concerns, misinformation and rumours can be addressed.

As the incidence of COVID-19 cases increases, it will at some point no longer be feasible, or efficient, to trace all contacts of confirmed cases, thus discontinuing active case finding. This will probably happen at different points in time in different countries. We characterise this as progression to *Scenario 3a: Widespread sustained transmission with increasing pressure on the healthcare system*. The objective would then shift from containment to mitigation, requiring substantial risk communication effort to ensure that the public knows how to respond in case of a suspected infection. Countries may consider the implementation of social distancing measures such as cancellation of conferences, cultural or sport events or the recommendation of teleworking or school closures in order to slow transmission of the virus. Such measures may reduce the acute burden on healthcare systems and possibly delay and/or reduce the peak of an outbreak. In this phase, it may be essential to simplify case reporting and test for SARS-CoV-2 in specimens from syndromic primary healthcare and hospital-based influenza surveillance systems. Detections of SARS-CoV-2 via influenza surveillance would initially be an indicator of transmission in the community and over time would allow the spread, intensity and severity of the virus to be described. Preparations should be made for efficient triage of cases requiring medical attention and for cohort isolation of cases requiring treatment. Citizens should be advised on what they can do to reduce pressure on the healthcare system.

The severity of COVID-19 remains unclear, but initial indications are that older adults and those with comorbidities are at higher risk [2]. If infection with SARS-CoV-2 becomes widespread, even a small proportion of severe cases could place healthcare systems under heavy pressure, resulting in *Scenario 3b: Widespread sustained transmission with overburdened healthcare system*. The burden will be compounded if the novel virus co-circulates with seasonal influenza, which stretches hospital capacity in many countries each winter. As at 28 February, seasonal influenza activity remains high in the majority of European countries but the peak of transmission seems to be past in several countries [4]. In the event that hospitals, emergency rooms, and intensive care units are unable to admit patients because of insufficient numbers of beds or staff, countries should be ready to implement contingency plans (e.g. adapt standard hospital beds for the treatment of severe cases). It may be necessary to reschedule non-essential operations and to evaluate whether alternative locations could be used to provide healthcare. Redistribution of resources throughout the EU/EEA could be considered.

## Conclusion

Today, much uncertainty remains around the ongoing epidemic and how it will unfold in the EU/EEA. Public health bodies and research institutions should continue to work together to continuously evaluate the situation, to address knowledge gaps and to assess the effectiveness of targeted interventions with new tests, treatments and vaccines as they are developed. Nevertheless, given the current knowledge and understanding, it is clear that action should be taken immediately to prepare for potential mitigation phases and coordinate efforts to protect the health of EU/EEA citizens in compliance with Decision 1082/2013/EU on serious cross-border threats to health [5]. As the number of cases in the EU/EEA is rapidly increasing, countries should reinforce their risk communication efforts and review their pandemic preparedness plans to ensure they are ready to avert or respond to more widespread circulation of the virus in the EU/EEA.
